# Assessing population‐based to personalized planning strategies for head and neck adaptive radiotherapy

**DOI:** 10.1002/acm2.14576

**Published:** 2024-12-03

**Authors:** Justin Visak, Chien‐Yi Liao, Xinran Zhong, Biling Wang, Sean Domal, Hui‐Ju Wang, Austen Maniscalco, Arnold Pompos, Dan Nyguen, David Parsons, Andrew Godley, Weiguo Lu, Steve Jiang, Dominic Moon, David Sher, Mu‐Han Lin

**Affiliations:** ^1^ Department of Radiation Oncology University of Texas Southwestern Medical Center Dallas Texas USA; ^2^ Medical Artificial Intelligence and Automation Laboratory Department of Radiation Oncology University of Texas Southwestern Medical Center Dallas Texas USA

**Keywords:** adaptive radiotherapy, head & neck, machine‐learning

## Abstract

**Purpose:**

Optimal head‐and‐neck cancer (HNC) treatment planning requires accurate and feasible planning goals to meet dosimetric constraints and generate robust online adaptive treatment plans. A new x‐ray‐based adaptive radiotherapy (ART) treatment planning system (TPS) version 2.0 emulator includes novel methods to drive the planning process including the revised intelligent optimization engine algorithm (IOE2). HNC is among the most challenging and complex sites and heavily depends on planner skill and experience to successfully generate a reference plan. Therefore, we evaluate the new TPS performance via conventionally accepted planning strategies with/without artificial intelligence (AI) and knowledge‐based planning (KBP).

**Methods:**

Our institution has a pre‐clinical release of the Varian Ethos2.0 TPS emulator which includes several changes that may affect current planning strategies. Twenty definitive and post‐operative HNC patients were retrospectively selected with a two or three‐level simultaneous integrated boost (SIB) dosing scheme. Patients were replanned in the emulator using population‐based, KBP‐guided with/without human intervention and AI‐guided planning goals. These planning strategies were compared both dosimetrically and for plan deliverability.

**Results:**

All strategies generally demonstrated acceptable plan quality with KBP‐ and AI‐guided goals offering enhanced dosimetric sparing in organs‐at‐risk (OAR). The average contralateral parotid gland mean dose was 20.0 ± 6.1 Gy (*p* < 0.001) for population‐based and 15.0 ± 6.1 Gy (*p* = n.s.) for KBP‐with human intervention versus 15.1 ± 7.4 Gy for clinical plans. Target coverage, minimum dose, and plan hotspot were acceptable in all cases. KBP‐enabled strategy demonstrated higher modulation and faster optimization time than both population‐based and AI‐guided strategies.

**Conclusion:**

Simply entering population, automatic KBP‐enabled or AI‐generated planning goals into the new Ethos2.0 TPS produced dosimetrically compliant plans, with AI‐guided goals demonstrating the most OAR sparing. Several of these approaches are easy to translate to other treatment sites and will help lower the barrier to entry for x‐ray‐based online‐ART.

## 1 INTRODUCTION

Radiotherapy treatment planning for head and neck cancer (HNC) is highly complex due to the proximity of several organs‐at‐risk (OAR) to high‐dose targets. Final plan quality heavily depends on a planner's skill, experience, and planning time.^1^, ^2^, ^3^ Clinics may deploy several different methods to efficiently generate plans of acceptable quality. In general, all treatment plan goals stem from population‐based metrics such as RTOG^4^ guidelines. With an increased presence of knowledge‐based planning (KBP) and artificial intelligence (AI) capabilities,^5^, ^6^ it is becoming common to use these tools to help improve plan quality, mainly by increasing OAR sparing and reducing inter‐planner variability.^6^, ^7^, ^8^ Online adaptive radiotherapy (oART) has recently emerged as a promising option for the treatment of various sites including complex head and neck radiotherapy, where a newly generated plan can be delivered based on daily imaging.^9^ Head and neck cancer patients uniquely may benefit from oART as standard treatment typically requires long‐course conventional fractionation. Patients often exhibit inter‐fractional, systematic anatomical changes and tumor shrinkage that may be accounted for by oART, and more aggressive daily oART schemes may facilitate potential margin reduction.^10^, ^11^

Online ART compresses the conventional planning timeline into less than one hour while the patient remains on the treatment table using either MR‐guided or x‐ray‐guided daily imaging.^12^, ^13^ This is typically accomplished with the use of AI, intelligent re‐optimization solutions, and streamlined software.^9^ A major focus of all the online ART platforms is to compensate for inter‐fractional changes with MR‐guided platforms offering superior tissue contrast and x‐ray‐based allowing for familiar CBCT imaging acquisition.^14^ Regardless of the ART‐platform, the success of HNC oART treatment hinges on the generation of a high‐quality initial reference plan that defines the online strategy in subsequent treatments. Reference planning for ART represents a paradigm shift in strategy as the planner must consider how the targets and OARs may evolve throughout the course of treatment. This significantly adds to an already high HNC planning burden and increases the technical skill required for robust reference plan generation. The requirement of specialized staff, equipment, and time resources may ultimately discourage the use of x‐ray‐based ART for HNC.^15^ Adaptive planning is also still relatively in its infancy compared to conventional techniques while early studies are beginning to provide more technical guidance of each respective platform.^16^, ^17^, ^18^ Therefore, integrating existing conventional automation tools into the adaptive reference planning workflow is crucial, as it helps improve treatment efficiency and reduce staff burden.

X‐Ray‐based ART currently lacks a clear and streamlined path to inject existing AI or KBP models into the workflow without manual transcription of model output into the treatment planning system (TPS).^19^ Allowing for AI/KBP guidance in reference planning could reduce the technical skill level required for high‐quality plan generation. The primary focus of this study is to dosimetrically evaluate reference planning techniques in the extremely complex HNC‐ART space utilizing several different planning techniques with and without AI and KBP assistance. We hypothesize that the new approach will generate clinically acceptable plans with an efficiency that may decrease the entry barrier to ART via streamlined approaches already available in conventional planning.

## 2 METHODS

### 2.1 Patient demographics and dosing schemata

Institutional Review Board (STU 082013‐008) approval was obtained for the use of all patient data included in this study. Twenty patients who were clinically treated at our institution with IGRT delivery were selected. Patients were immobilized using a 5‐point thermoplastic mask that covered the top of the head to the upper chest. All clinical cases were optimized in Eclipse TPS with beam geometry normally using either 12–18 IMRT or 4–5 arc VMAT fields typically utilized in our clinic. To represent a wider patient cohort, ten patients that were treated with definitive RT and ten with post‐operative RT were selected for this study typically treated using a three‐dose level simultaneous integrated boost (SIB) approach with dose 70 Gy in 33–35 fractions and two‐dose level SIB 60 Gy in 30 fractions, respectively. All PTVs used conventional standard‐of‐care margin expansion (3–5 mm from target structures). All plans are normalized such that 95% of the highest PTV level receives 100% of the prescription dose.

### 2.2 Treatment planning strategies

**FIGURE 1:**
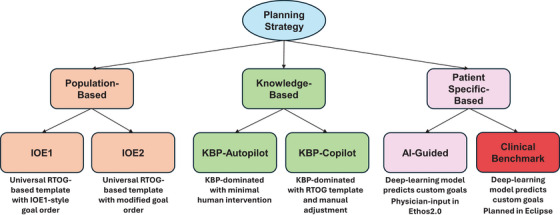
Summarized flowchart of HNC planning strategies. General planning strategy ranging from population to patient‐specific‐based methods. IOE1 and IOE2 plans utilized universal RTOG templates with differing goal orders. Knowledge‐based techniques evaluate the influence on optimization with or without human intervention. The patient‐specific‐based strategy utilized custom AI‐based planning goals with physician input including clinically treated cases guided by the same AI objectives. AI, treatment planning system; HNC, head‐and‐neck cancer; IOE, intelligent optimization engine.

Our institution has pre‐clinical access to the new Varian Ethos 2.0 (Ethos2.0) TPS emulator (Varian Medical Systems, Palo Alto, USA). We aimed to evaluate all clinically available approaches ranging from population‐based to patient‐specific based strategy in the Ethos2.0 emulator and benchmark to the clinically treated cases. Each approach is detailed later and summarized in Figure 1. All newly generated plans were normalized to have identical coverage to their clinical counterparts and utilized a standard IMRT ART beam geometry template. A generalized HNC planning strategy has been presented elsewhere for the currently available Ethos TPS.^19^ The primary goal of these selected planning strategies is to investigate if current recommendations require modification to account for changes in the underlying optimization algorithms of the new TPS along with the evaluation of AI/KBP enhancements.

#### 2.2.1 Population‐based template planning strategy

The first group of planning strategies deployed to guide the new Ethos2.0 utilized a simple and robust universal template where OAR clinical goals were based on RTOG reports.^19^ In the current Ethos TPS implementation, the system treats all user‐defined priority goals (P1‐P4) goals of similar importance whereas Ethos2.0 now widens the strength of each level during optimization and potentially represents a more intuitive planning rationale. Supplemental goals such as hotspot controlling, plan complexity, and automatically generated ring structures to decrease intermediate dose spillage have also been reworked to increase priority against the planner's input goals. To isolate these changes, two plans were generated in Ethos2.0 and share identical clinical OAR goals as defined by the RTOG dosing criteria. The first plan, IOE1, utilized a goal layering order identical to the strategy utilized in the current clinical TPS system whereas the other population‐based plan IOE2 deploys a revised layering strategy as the new Ethos2.0 can accept templates from the current clinical system. The fundamental difference between the IOE1 and IOE2 plans is the priority level of clinical goals as the Ethos2.0 changes indicate it is beneficial to spread out clinical goals between priority levels.

#### 2.2.2 Knowledge‐based model planning strategy

**FIGURE 2:**
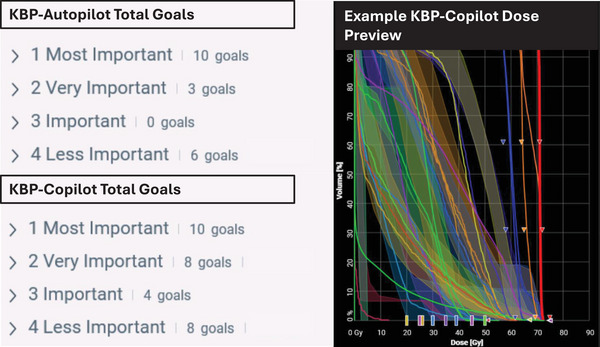
Example of total clinical goals of KBP‐autopilot and copilot approach. Most clinical goals ranked in priority 1 for both autopilot and copilot approaches are reserved for target coverage and fall‐off. In general, these goals are fixed order for all patients, and priorities 2 & 3 are reserved for OARs that planner iteratively uses to drive optimization. Note that autopilot approach has minimum goals and is primarily driven by the KBP‐DVH estimation algorithm. KBP, knowledge‐based planning; OAR, organs‐at‐risk.

The priority of KBP models has increased, (bottom of Priority 2) and now has the potential to significantly impact the final plan quality. Previously, the use of KBP was limited as user input goals dominated over DVH‐estimation. Two strategies were used to help isolate the influence of our in‐house trained KBP model. The first strategy, KBP‐autopilot, aimed to allow the KBP model to effectively drive the entire plan optimization process with minimum human intervention as all clinical goals besides target and serial maximum dose objectives were removed. By reducing the total number of goals, optimization control is shifted towards automation by the KBP‐model. Alternatively, KBP‐copilot deployed the KBP model in conjunction with the existing RTOG universal dosing template (e.g., planner assumes more optimization control with increased burden). The additional goal order was tweaked to maximize the IOE2 changes and physician‐defined trade‐offs. An example goal and KBP‐DVH estimation preview is shown in Figure 2 to demonstrate the reduction of OAR goals utilized in autopilot setting from approximately 30 iterative planning objectives to 19. Typically, P1/P4 goals contain target or tuning structures.

#### 2.2.3 AI‐based planning strategy

Our institution utilizes a 3D dose prediction deep learning algorithm to help guide the physician and planner to generate a patient‐specific template for HNC ART cases.^6^, ^20^ The physician is presented with an idealized 3D dose distribution with corresponding OAR dosing criteria. In a streamlined process, the physician will then tweak and modify this suggested criterion and communicate with the planner. For the current version of TPS, these communicated planning criteria are then transcribed manually into the TPS and serve as the initial planning template and priority is iteratively adjusted. In this study, all clinical cases were planned with AI‐guidance in the conventional TPS. The exact planning criteria used for the clinical plans were input directly into Ethos2.0. This allowed the new IOE2 to optimize solely on the AI‐predicted dose values.

### 2.3 Dosimetric and plan deliverability metrics

All plans generated in the novel x‐ray‐based ART TPS were compared against their corresponding clinical plans. In addition to high‐impact OAR compliance, target coverage, and plan hotspots were assessed. Specifically, plans were evaluated as to whether they comply with RTOG‐based OAR dose limits while maintaining target coverage. Target coverage quality and hotspot for the highest and lowest PTV levels were assessed as all plans minimally utilized a two‐dose level SIB planning technique. Ethos2.0 provides advanced metrics beyond total monitor unit calculation to help the planning team assess plan quality. Herein, we recorded these additional multi‐leaf collimator (MLC) metrics and optimization time to help provide a holistic view of the plan beyond simple dosimetric evaluation. The advanced metrics utilized in this study were: Modulation complexity score (MCS), which assesses plan modulation by analyzing segment variability, shape, and area, a higher MCS value indicates a more complex field^21^; small aperture score (SAS10), which calculates the proportion of open MLC leaf pairs with an opening less than 10 mm; and Penumbra ratio, calculates the proportion of the aperture area close to the aperture boundary.^22^ To add clinical significance to these results, Monte‐Carlo in‐house second calc‐based QA software with 3%/2 mm gamma analysis criteria was used in this study.^23^ In our analysis, we utilized the Kruskal‐Wallis H Test to evaluate whether there are statistically significant differences across the independent groups. This non‐parametric test was particularly chosen because nearly half of our data did not follow a normal distribution, necessitating an alternative to traditional parametric methods like ANOVA. Upon identifying significant differences with the Kruskal–Wallis test, we conducted a further analysis using Dunn's post‐hoc test with Bonferroni correction to perform pairwise comparisons between the groups. The Bonferroni correction was specifically utilized to adjust the p‐values for multiple comparisons, thereby effectively controlling the family‐wise error rate and substantially minimizing the risk of Type I errors.

## 3 RESULTS

### 3.1 Target metrics

**FIGURE 3:**
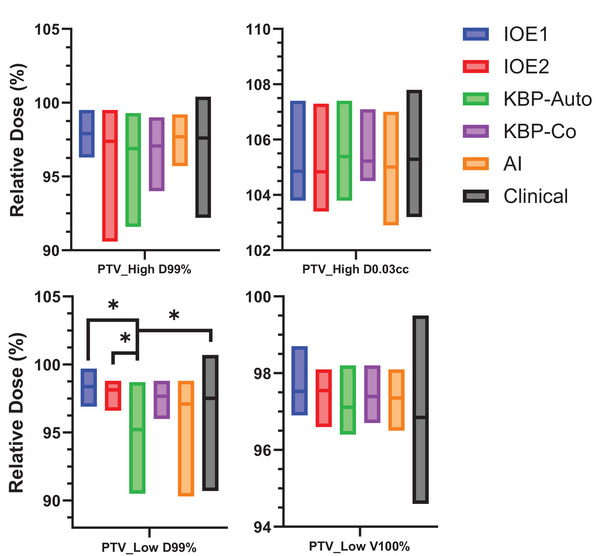
Evaluation of target metrics for highest and lowest SIB targets. Plan hotspot was assessed using the maximum dose the highest level PTV. All plans generated provided acceptable hotspots. Additionally, PTV was low near minimum dose, and coverage was maintained for all plans with respect to clinically treated plan. Statistical significance is denoted by *. SIB, simultaneous integrated boost.

In general, the plan hotspot was clinically acceptable and on average < 108% (range: 103%–108%) of prescription dose in all plans as seen in Figure 3. PTV maximum hotspot for IOE2, KBP‐autopilot, and KBP‐copilot was 104.8% ± 0.75%, 105.4% ± 0.78%, and 105.2% ± 0.61%, respectively. To assess the minimum dose and shoulder, a dose of 99% of the volume (D99%) was analyzed for both the highest and lowest PTV levels, and all plans were found to be clinically acceptable. In general, differences in target minimum dose were best displayed in the lowest PTV level as seen in Figure 3. The population‐based and AI‐guided cases yielded lower values on D99% and prescription coverage likely due to more aggressive OAR sparing. On average, all mean lower target prescription coverage exceeded 95%.

### 3.2 OAR sparing

**FIGURE 4:**
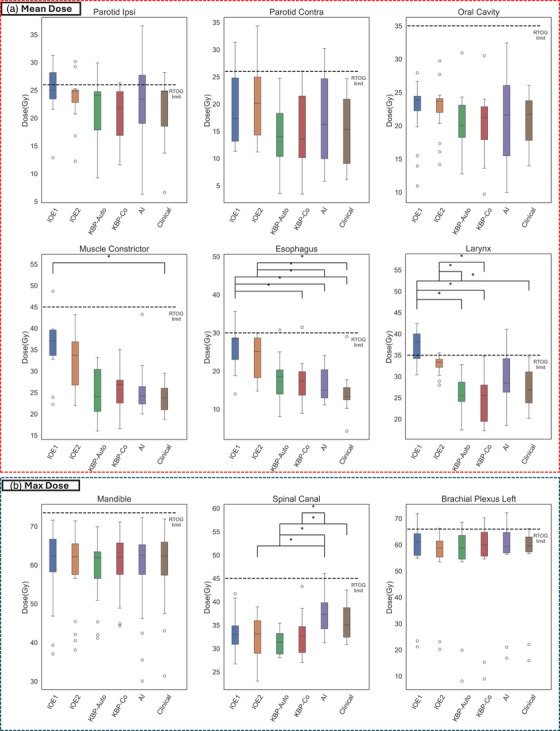
Evaluation of OAR mean and maximum dose criteria. Mean dose criteria (a) and maximum dose criteria (b) were evaluated for all OAR. Note that population‐based methods typically met RTOG criteria, whereas KBP‐enabled and AI‐dose‐guided plans provided more OAR sparing with respect to population‐based and clinically treated plans. Additionally, the enhanced strength of the KBP in the new TPS is shown in the mean dose as it more closely aligns with the AI‐guided planning strategy. Statistical significance is denoted by *. AI, artificial intelligence; KBP, knowledge‐based planning; OAR, organs‐at‐risk.

Several high‐impact OARs with corresponding statistical significance are displayed in Figure 4 which include both mean doses (red) and D0.03cc maximum dose (blue). On average, all OARs reported in this study safely met the RTOG limit with a few outlier exceptions (e.g., ipsilateral parotid for bilateral low dose target). Using the population‐based approach in IOE1 and IOE2‐style plans yielded higher OAR doses than alternative approaches and the clinical benchmarked plans. With minor goal order modification, the esophagus mean dose is reduced on average 2.2 ± 2.3 Gy (*p* = n.s.) between the IOE2 and IOE1‐style cases.

The knowledge‐based plans yielded lower OAR doses than population‐based and demonstrated an increased influence of the KBP model compared to prior studies. While the KBP‐autopilot which required minimal human intervention showed better OAR sparing than population‐based plans, the KBP‐copilot cases with human intervention were closer to the AI clinical benchmark cases. For example, while IOE2 produced an average contralateral parotid gland mean dose of 20.0 ± 6.1 Gy (*p* = n.s.), the KBP‐copilot and clinically treated plan was 15.0 ± 6.1 Gy (*p* = n.s.) and 15.1 ± 7.4 Gy, respectively. The most well‐balanced plan for OAR sparing was observed in the AI‐guided clinical and AI‐based Ethos2.0 plans demonstrating closest resemblance to clinical plan trade‐offs.

**FIGURE 5:**
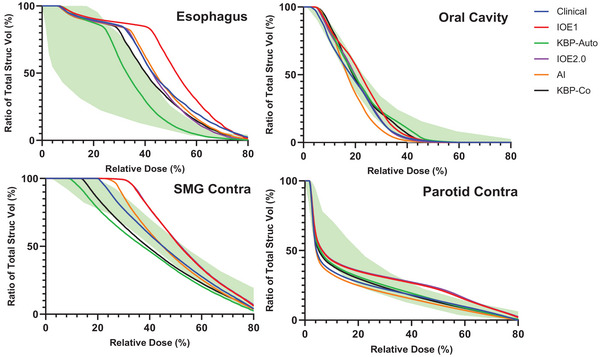
Evaluation of OAR sparing tradeoffs. The esophagus, oral cavity, contralateral submandibular gland, and parotid gland were selected and plotted for all planning strategies using a single example patient. The green band represents the DVH estimate provided by the KBP model. Note that each strategy prioritizes different levels of OAR sparing. KBP, knowledge‐based planning; OAR, organs‐at‐risk.

To isolate the effect of the KBP influence in Ethos2.0, a definitive case was selected for enhanced analysis. The example case chosen utilized a three‐dose level technique of 70, 63, and 56 Gy, respectively. All target coverage, OAR sparing and plan hotspots were acceptable. Four OARs, esophagus, oral cavity, contralateral submandibular and parotid gland were selected and DVHs replotted in Figure 5 against the KBP model DVH‐estimation (green shading). Each strategy handles OAR sparing differently and is based on institutional/patient‐specific criteria. For example, the KBP‐autopilot preferentially spares the esophagus dose whereas the physician input AI‐guided plan favors the oral cavity.

### 3.3 Plan deliverability and optimization time

Plan deliverability metrics and total plan optimization time are reported in Table 1. This includes gamma‐analysis for all plans included in this study. Population‐based IOE1 and IOE2 plans on average demonstrated the lowest total monitor units and were statistically significant with respect to the AI‐guided plans. Alternatively, the KBP‐enabled strategies exhibited the highest modulation as evident in the total MU and advanced MLC‐based metrics. This is due to the KBP model uniformly pushing the entire DVH curve down (e.g., low‐dose wash). Interestingly, this added modulation does not affect total optimization time as the KBP‐guided plans converged quicker than other approaches. It is demonstrated that the KBP‐autopilot plan with minimal human intervention converges approximately 24 s quicker than AI‐guided. Despite differences in plan modulation, all planning approaches rendered clinically acceptable patient‐specific QA passing rates with a 3%/2 mm criteria. While the KBP‐enabled approach did show a slightly decreased and statistically significant pass rate, all plans passed well above 95%.

**TABLE 1:** Plan deliverability metrics.

Plan type	Total MU	γ Pass rate 3%/2 mm (%)	MCS	SAS10 (%)	Penumbra width (1/mm)	Optimization time (s)
Clinical	2371 ± 423 (1550–3179)	99.2 ± 1.2 (95.2–99.3)	–	–	–	–
IOE1	2076 ± 263 (1558–2471)	99.2 ± 0.4 (98.6–99.8)	0.90 ± 0.01 (0.87–0.92)	33.8 ± 2.7 (29.8–42.5)	52.3 ± 2.5^*^ (49.1–61.1)	181.0 ± 27.8 (134.7–247.5)
IOE2	2122 ± 262 (1661–2523)	99.2 ± 0.3 (98.5–99.7)	0.90 ± 0.01 (0.87–0.92)	34.7 ± 2.7 (30.3–40.5)	52.8 ± 2.0 (49.7–58.1)	180.7 ± 27.2 (137.2–247.9)
KBP‐Auto	2277 ± 175 (1844–2559.1)	99.0 ± 0.4 (98.0–99.7)	0.91 ± 0.01 (0.88–0.92)	37.3 ± 3.7 (32.2–48.0)	55.5 ± 3.8 (50.2–65.3)	157.8 ± 27.6 (111.1–214.0)
KBP‐Co	2322 ± 167 (1960–2570)	98.9 ± 0.4 (97.8–99.7)	0.90 ± 0.009 (0.89–0.92)	37.4 ± 4.1 (30.2–48.3)	55.9 ± 3.8 (50.4–65.3)	175.2 ± 29.9 (124–247.1)
AI‐Guided	2233 ± 229 (1704–2520)	99.2 ± 0.4 (98.2–99.7)	0.90 ± 0.01 (0.88–0.92)	36 ± 2.6 (31.6–42)	54.5 ± 2.5 (50–59.6)	181.9 ± 27.8 (140–253.6)

* Denotes statistical signficance to AI‐guided plans.

*Note*: Analysis of plan delivery metrics for all cases utilized in this study. Note that while KBP‐enabled strategies created the most modulated plans, they on average required less optimization time with KBP‐autopilot generating the quickest plan.

Abbreviations: IOE, intelligent optimization engine; MCS, modulation complexity score; SAS, small aperture score.

## 4 DISCUSSION

Online ART is an actively investigated treatment paradigm for HNC and many other treatment sites. It is generally more resource demanding to generate a robust reference plan as the planning strategy must account for potential inter‐fractional changes.^24^ The use of KBP/AI to improve treatment planning dosimetry an efficiency has been extensively studied in literature for conventional planning/existing Ethos1.1 TPS.^25^, ^26^ Specifically, this study expands Visak et al.^19^ work that previously demonstrated KBP predicted goals in Ethos1.1 version did not significantly impact optimization or provide patient‐specific customization by making a direct comparison to conventional plans with AI goals. It was noted prior integration of KBP did not push the dosimetric goals to their best achievable values. However, we provide direct evaluation of the newly modified KBP algorithm incorporation that has yet to be validated for various treatment sites to help assess which potential planning strategy suits institutional goals. This study to our knowledge is the first to evaluate practical treatment planning strategies in the novel Varian Ethos2.0 for head and neck cancer treatments with/without the integration of KBP/AI which inserts the predicted KBP goal to a priority 2 weighting. This study indicates that all approaches will yield an RTOG compliant plan in most cases. However, the introduction of KBP/AI will increase overall plan quality and help lower the burden of complex HNC treatment planning. Institutions interested in x‐ray‐based ART for HNC patients should evaluate which approach best suits their clinical needs. The general planning strategy for all techniques is presented in Appendix A. Each planning strategy described in this study offers unique advantages and limitations within Ethos2.0. Institutions already utilizing a population‐based approach should see similar plan quality to their current practice with robust templating. However, the KBP‐ and AI‐enabled approaches will allow for more OAR sparing at minimal costs. The KBP strategies presented in this manuscript allow different levels of KBP integration within the optimization process. Utilizing the KBP‐autopilot increased planning autonomy but allows the system to automatically evaluate OAR/target tradeoffs whereas the KBP‐copilot method increases control of the optimization and the expense of efficiency. Regardless, Ethos2.0 effectively increased the power of a KBP model and its ability to drive optimization.^19^ The KBP‐model predicted objectives are converted to goals and placed in the bottom of the dose preview priority 2 group. The OAR sparing will also be driven by the layering order. It is important to note that generating a high‐quality HNC plan using KBP/AI assistance is extremely dependent on the quality of the prediction model.^27^ Our models were trained with similar patient data that was highly curated by AI experts. This helps to maximize plan quality and the utilization of the tools (e.g., KBP‐integration) provided in the Ethos2.0 system.

Other studies in literature have demonstrated that both autonomous and manually template‐based planning is suitable for ART. For example, Tengler et al. describes a fully automated planning method that utilizes a particle swarm optimization technique for 1.5T MR linac reference planning for prostate cancer that increases plan compliance with institutional criteria.^28^ Their study did not require human supervision which significantly decreases planner burden; however, particle swarm optimization may lead to increased total optimization times. Alternatively, our semi‐autonomous methods described in this work require more frequent human checkpoints in plan optimization but still can help to increase planner efficiency from template‐based approaches. Another study from Nasser et al. investigated template‐based planning approaches in the current x‐ray‐based ART planning system for HNC and found that in general plan quality is acceptable. Our study agrees with their work utilizing the new Ethos2.0 system and further expands alternative approaches that allow plans to be pushed for further dosimetric sparing with the use of KBP/AI.^29^

One major limitation of this study is that these planning strategies have not yet been evaluated for online robustness via emulation. Previous studies have shown that with a high‐quality robust reference plan, online replanning is feasible and efficient, so it is reasonable to infer these planning strategies will produce similar results online.^30^, ^31^ This study also recorded advanced plan delivery metrics to provide more technical plan information. In the future, this information may be useful to help predict treatment deliverability/improve QA practice (e.g., gamma pass rate vs. MCS) to improve overall treatment confidence. Furthermore, the use of static templates generated via patient‐specific‐AI or institution criteria in oART may limit plan quality by disallowing dynamic optimization with the current system configuration. An interesting future study would be to evaluate how well the KBP influence can help create a more dynamic optimization online as the DVH‐estimation can be recalculated online.^32^ This study serves as an important first step toward demonstrating an automatic dynamic optimization planning solution.

## 5 CONCLUSION

In summary, HNC poses challenges in x‐ray‐based ART reference planning, highlighting the need for a simplified, uniform methodology. The assessed strategies consistently produce plans that meet dosimetric requirements, potentially facilitating wider adoption of x‐ray‐based online ART. Utilizing KBP and AI will further refine plans, optimizing them to balance patient‐specific considerations for enhanced quality.

## AUTHOR CONTRIBUTIONS

Justin Visak and Mu‐Han Lin designed the study. Justin Visak, Chien‐Yi Liao, and Mu‐Han Lin performed a replanning of clinical cases. Biling Wang, Sean Domal, Hui‐Ju Wang, Austen Maniscalco, and Dan Nyguen performed data collection, model development, or statistics. Weiguo Lu provided software for this project. Justin Visak and Mu‐Han Lin drafted the initial version of the manuscript. Arnold Pompos, Andrew Godley, Steve Jiang, Dominic Moon, and David Sher provided clinical supervision and input to the project. All authors revised and approved the final manuscript.

## CONFLICT OF INTEREST STATEMENT

The authors declare no conflicts of interest.

## ETHICS STATEMENT

Institutional Review Board approval was obtained for the use of all patient data included in this study (STU 082013‐008).

## Supporting information



Supporting Information

## Data Availability

The data that support the findings of this study are available from the corresponding author upon reasonable request.
