# Supplementary material for: Assessing population‐based to personalized planning strategies for head and neck adaptive radiotherapy
Source: J Appl Clin Med Phys. 2024 Dec 3;26(3):e14576. doi: 10.1002/acm2.14576 (PMC11905247; doi:10.1002/acm2.14576)
Supplement: Supplementary file 1 — Supporting Information [file ACM2-26-e14576-s001.docx]

**Appendix A**

| **Generalized Planning Strategy for X-ray Based H&N Adaptive Radiotherapy Planning.** *Updated generalized planning strategy for robust HNC adaptive planning utilizing novel x-ray based ART. Note the more intuitive planning approach that fully utilizes all priority order levels in Ethos2.0. The optPTV/dose-tuning structure is primarily used to control intermediate dose-leakage to lower PTV levels.* | | | |
| --- | --- | --- | --- |
| **Structure** | **Goal** | **Order** | **Formula/Note** |
| PTV_D_max_ | D_0.03cc_ < 107% Rx | Highest P1 | (All PTV + 2 cm) minus body |
| Hard Constraint OAR | MD Defined Clinical Goal | P1 | -- |
| PTV | V_100%_ ≥ 95% | P1 | -- |
| Serial Structures | MD Defined Clinical Goal | P1 | -- |
| PTV | V_95%_ ≥ 99% | P1 | -- |
| High-Impact OAR | MD Defined | P2 | -- |
| OAR spared > 5 Gy Goal | MD Defined | P3 | -- |
| Tier 2 Parallel OAR | MD Defined | P3 | -- |
| OAR violating > 5 Gy Goal | MD Defined | P4 | -- |
| optPTV/dose-tuning | D_0.03cm3_<110% RXlow | P4* | [PTV_low_ – (PTV_high_+ x mm)] |
|  | D_30%_< Rxlow + 1 Gy  D_60%_< Rxlow + 2 Gy |  | x= (Rx_high_-Rx_low_)/3 + 1 mm |

***At institution discretion**
